# Efficient Brain Tumor Detection with Lightweight End-to-End Deep Learning Model

**DOI:** 10.3390/cancers15102837

**Published:** 2023-05-19

**Authors:** Mohamed Hammad, Mohammed ElAffendi, Abdelhamied A. Ateya, Ahmed A. Abd El-Latif

**Affiliations:** 1 EIAS Data Science Lab, College of Computer and Information Sciences, Prince Sultan University, Riyadh 11586, Saudi Arabia; affendi@psu.edu.sa (M.E.); aateya@psu.edu.sa (A.A.A.); 2 Department of Information Technology, Faculty of Computers and Information, Menoufia University, Shibin El Kom 32511, Egypt; 3 Department of Electronics and Communications Engineering, Zagazig University, Zagazig 44519, Egypt; 4 Department of Mathematics and Computer Science, Faculty of Science, Menoufia University, Shebin El Koom 32511, Egypt

**Keywords:** Internet of Medical Things, CNN, brain tumor detection, deep learning, security

## Abstract

**Simple Summary:**

This paper discusses the importance of early detection of brain tumors and the limitations of traditional diagnosis methods. The use of deep learning models for brain tumor detection is introduced as a potential solution, but the high computing costs and potential biases in training data pose challenges. The study proposes a new, end-to-end, lightweight deep learning model for brain tumor detection that outperforms other models and is suitable for real-time applications. The study also provides a framework for secure data transfer of medical lab results and security recommendations to ensure security on the Internet of Medical Things (IoMT).

**Abstract:**

In the field of medical imaging, deep learning has made considerable strides, particularly in the diagnosis of brain tumors. The Internet of Medical Things (IoMT) has made it possible to combine these deep learning models into advanced medical devices for more accurate and efficient diagnosis. Convolutional neural networks (CNNs) are a popular deep learning technique for brain tumor detection because they can be trained on vast medical imaging datasets to recognize cancers in new images. Despite its benefits, which include greater accuracy and efficiency, deep learning has disadvantages, such as high computing costs and the possibility of skewed findings due to inadequate training data. Further study is needed to fully understand the potential and limitations of deep learning in brain tumor detection in the IoMT and to overcome the obstacles associated with real-world implementation. In this study, we propose a new CNN-based deep learning model for brain tumor detection. The suggested model is an end-to-end model, which reduces the system’s complexity in comparison to earlier deep learning models. In addition, our model is lightweight, as it is built from a small number of layers compared to other previous models, which makes the model suitable for real-time applications. The optimistic findings of a rapid increase in accuracy (99.48% for binary class and 96.86% for multi-class) demonstrate that the new framework model has excelled in the competition. This study demonstrates that the suggested deep model outperforms other CNNs for detecting brain tumors. Additionally, the study provides a framework for secure data transfer of medical lab results with security recommendations to ensure security in the IoMT.

## 1. Introduction

Brain tumors are a group of tumors that develop in the structures of the central nervous system (CNS). They can be benign or malignant. Some diseases are completely cured, while, in others, the life expectancy of a person after diagnosis is only 2–3 years [1]. The World Health Organization (WHO) classifies BTs into four categories (Grade I–IV) depending on their malignancy or benignity. The standard approaches for detecting and analyzing BTs are magnetic resonance imaging (MRI) and computer tomography (CT) [2]. Early diagnosis of a brain tumor is a dilemma. After all, with small tumor sizes, the disease does not manifest itself clinically. Accordingly, the patient does not consult a doctor. Diagnosing a brain tumor may be delayed when it reaches a large size. In fact, when the location of the tumor is in functionally active parts of the nervous system or in a deep location, the tumor may be inoperable, or the results of the surgery may cause significant neurological deficits [3]. Therefore, detecting these kinds of tumors early is vital to reduce the symptoms of these tumors.

Often, the clinical diagnosis of brain tumors includes finding these signs of the disease: motor coordination disorder, signs of cranial nerve damage, signs of intracranial hypertension, epileptic seizures, paresis (weak muscle tone), speech disorders, etc. Such signs can be detected by a physician, neurologist, or attending physician. Later, if a brain tumor is suspected, the diagnosis is made by magnetic resonance imaging and biopsy. Brain metastasis cannot be detected based on objective symptoms alone. However, it is possible to determine the level of damage to the CNS as well as the severity of the patient’s condition. This is important for determining the timing of radical treatment. Then follows the instrumental diagnosis of brain tumors [4].

The most common method is MRI, which is currently the best way to detect a brain tumor in anyone with unexplained neurological symptoms. This method makes it possible to identify a brain tumor 2 mm in size or larger. MRI also allows to determine the exact localization of the tumor, infer the type of histological tumor, assess whether there is edema and whether the structure of the brain has changed, determine the location of the tumor in relation to the main vessels (the introduction of contrast material is required), and determine the location of the tumor in relation to the functionally active areas of the brain, by functional MRI. The most common types of intracranial tumors are high-grade glioma, low-grade glioma, meningioma, and pituitary adenoma [5]. The relevance of tumor structures in different image modalities is a crucial consideration in brain tumor detection and segmentation using MRI images. Different MRI modalities, such as T1, FLAIR, and T1c, provide complementary information about the tumor’s location, size, and morphology. T1-weighted images provide excellent contrast between gray and white matter, making them suitable for identifying the tumor’s location. FLAIR images suppress the signal from cerebrospinal fluid (CSF) and allow for better visualization of edema and tumor infiltration in surrounding brain tissue. T1c images, which are T1-weighted images acquired after the injection of a contrast agent, provide better visualization of the tumor’s enhancement, indicating the presence of active tumor tissue.

In the proposed methodology, we employed T1, T2, and FLAIR images to classify brain tumors into different categories. However, recent studies have shown that combining multiple MRI modalities can lead to more accurate segmentation and classification of brain tumors. For instance, a study by Zhao et al. [6] used a deep learning-based segmentation approach that combined T1, T1c, T2, and FLAIR images to segment brain tumors more accurately than using each modality alone. Similarly, a recent study by Zhang et al. [7] presented a multimodal deep learning approach that combined T1, T2, and FLAIR images to improve the detection and classification of brain tumors. Therefore, it is essential to consider the relevance of tumor structures in different image modalities when developing a brain tumor detection or segmentation algorithm. Combining multiple modalities can provide complementary information and improve the accuracy of the algorithm.

According to previous researchers, machine and deep learning approaches using MRI data could lead to easier detection and classification of brain tumors [8,9,10,11,12,13,14,15,16,17,18,19]. However, these approaches suffer from the following limitations:

- Most of these methods have high computational costs and require large amounts of labeled data for training.
- Potential for overfitting, where the model performs well on the training data but not on new, unseen data.
- Risk of poor performance due to biased training data or incorrect labeling.
- Lack of interpretability of the decision-making process of deep learning models.

In order to avoid overfitting or misdiagnosis, it is important to use robust and validated deep learning models that have been trained on large and diverse datasets. Additionally, it is important to consider potential biases in the training data and to use techniques such as cross-validation to ensure the model’s generalizability. Overall, the goal of our detection method is to improve patient outcomes by providing accurate and timely diagnoses, enabling prompt and effective treatment. This study addresses the most common intracranial tumors and determines tumor classes or no tumor from MRI images based on a deep learning approach. The proposed method is end-to-end, which makes the model less complex than other deep models. In addition, the proposed model is lightweight, as it consists of only eight layers, which makes the system suitable for mobile applications and other real-time applications. We can summarize the novelty of our work as follows:

- Propose a new end-to-end structure CNN model for detecting brain tumors. The proposed model can detect the most common intracranial tumors, such as high-grade glioma, low-grade glioma, meningioma, and pituitary adenoma, with acceptable accuracies compared to other previous deep models.
- Propose a new, lightweight deep learning model for brain tumor detection. Our model consists only of eight layers in depth, which makes the system suitable for real-time applications and reduces the time of processing, unlike other previous models in this field that needed deeper layers to obtain good detection accuracy.
- We tested our model on different datasets for brain tumors using the cross-validation technique, which overcame the overfitting problem and achieved good accuracy for the detection. In addition, we tested our model in different cases, such as binary classification and multi-class classification, which makes the model more robust than other previous models.

## 2. Related Studies

There has been a significant amount of research in the field of brain tumor detection, including both traditional computer vision techniques [8,9,10,11,12,13] and deep learning-based approaches [17,18,19,20,21,22]. Traditional computer vision techniques for brain tumor detection often involve manual feature extraction and classification using techniques such as support vector machines (SVM), decision trees, and Random Forest [23,24]. In recent years, deep learning techniques such as convolutional neural networks (CNNs) have become increasingly popular for brain tumor detection due to their ability to automatically learn features from medical imaging data [25,26,27]. These models have shown promising results in detecting and segmenting brain tumors, as well as in classifying different types of tumors. Several research studies have also explored the use of transfer learning, where a pre-trained deep learning model is fine-tuned on a smaller dataset specific to brain tumor detection [18,19,20,21].

Vankdothu et al. [14] presented an algorithm based on CNN combined with Long Short-Term Memory (LSTM) for brain tumor identification. They used traditional computer vision techniques for preparing the MRI images to be suitable for the classification stage. Therefore, they performed the pre-processing and feature extraction stages using traditional methods and the classification using the introduced deep learning model. They obtained an overall accuracy of 92% for the multi-class task to classify the input MRI images into normal, glioma, meningioma, and pituitary adenoma. Aamir et al. [15] introduced a deep learning model for classifying three classes of brain tumors using MRI images. They performed a pre-processing stage on the input images before feeding them to the deep model. After that, they extracted the features for all pre-processed images using a combination of two deep models and concatenated the features to obtain the final features. Then, they applied the generation algorithm to these hybrid features to generate the locations and ROI alignment. Finally, they fed the results to the remaining layers of the deep models for classification and detection. They obtained an overall classification accuracy of 98.83% using 40 epochs. Hashemzehi et al. [16] used a combination model between CNN and neural autoregressive distribution estimation (NADE) for the detection of brain tumors. The input images were fed directly to the presented CNN called CNN2 and NADE in parallel. The output of the NADE was fed into another CNN called CNN 1. Finally, the outputs of CNN1 and CNN2 were combined using fully connected layers to obtain the final detection of the MRI images into three classes. They obtained an overall accuracy of 95% after applying the six-fold cross-validation technique. Chattopadhyay and Maitra [17] presented a CNN method to detect brain tumors from MRI images. They fed the input images directly to the present CNN model for segmentation and feature extraction. Finally, they used a support vector machine as a separate classifier for final classification. They performed a binary classification task to classify the input images into normal and abnormal images and obtained an overall accuracy of 99.74%. Younis et al. [18] introduced a method using a combination of VGG-16 and ensemble learning approaches with a CNN model for the detection of brain tumors. They first performed on the input MRI images several image processing stages, such as image pre-processing, image augmentation, and image segmentation. After that, they fed the pre-processed images to the ensemble model, which consists of two deep models (CNN and VGG-16 models). Finally, they performed the prediction based on the results of the combination of the two models to distinguish between the tumor and healthy images with an accuracy of 98.41%. Rasool et al. [19] presented a combination model based on deep learning and SVM for the multi-classification of brain tumors. They fed the MRI input images directly to the presented deep pre-trained model (Google-Net) for extracting the features. After that, they fed the extracted features to the SVM for classification of the input images into normal and the other three classes. They obtained an overall accuracy of 94.12% using this combination between Google-Net and SVM.

From the previous discussion, we can observe that most of these previous works used combination models that make the system more complex and time consuming, as in [14,15,16,17,18,19]. In addition, most of these papers used traditional approaches combined with their deep model, such as using pre-processing in [14,15,18], while others used separated classifiers such as SVM, as in [17,19], and others used traditional feature extraction methods combined with their deep model, as in [15,16]. Finally, all of these studies obtained very low accuracy on multi-class tasks, and most of them suffer from overfitting and may have skewed findings due to inadequate training data. However, recent advancements in deep learning have addressed this issue by using techniques such as data augmentation and unsupervised learning to train models on larger and more diverse datasets [28,29,30,31,32,33,34]. Our method is more robust than all previous methods and achieves higher accuracy than these methods, especially in multi-class tasks and using a relatively small dataset. In addition, our model is a lightweight end-to-end method, unlike the other previous models, which were deeper and more complex.

## 3. Methodology and Datasets

**Figure 1:**
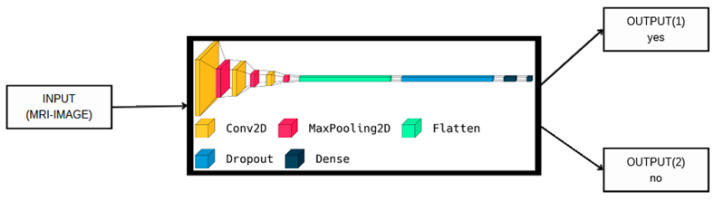
The proposed model visualization for binary class.

**Figure 2:**
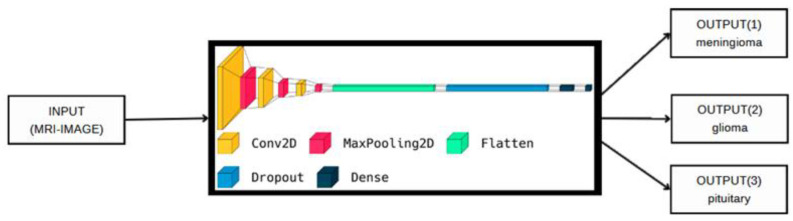
The proposed model visualization for multi-class.

In this section, the datasets used in this paper are discussed in detail. In addition, every stage of the proposed methodology is also discussed in detail. Figure 1 and Figure 2 show the block diagram of the proposed deep method for binary class and multi-class. In Figure 1, the input MRI images are fed directly to the proposed deep model to perform the final decision (yes if the image has tumor detection and no if the image is a normal image). The objective of this classification model is to accurately differentiate between the two classes based on the MRI scan images. In addition, the same scenario was applied to the input MRI images for the multi-classification problem, as shown in Figure 2. In this case (multi-class), the input images are classified into three classes, i.e., meningioma (output 1), glioma (output 2) and pituitary tumors (output 3). The input MRI images in both cases are drawn from two publicly available brain tumor datasets [22,23], which are discussed in detail in the following subsection.

### 3.1. The First Dataset

**Figure 3:**
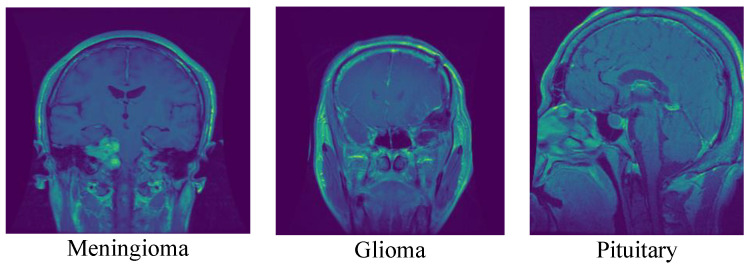
Samples of the three classes from the first dataset.

A total of 3064 T1-weighted contrast-enhanced MRI images from this dataset [22] have had the presence and location of brain tumors manually annotated by qualified radiologists. The images in the dataset have been acquired from 233 patients with different types of brain tumors, including glioma, meningioma, and pituitary tumors. The data are divided into two subsets: the training dataset, which contains 2204 images, and the testing dataset, which contains 860 images. The images are in 2D and have a resolution of 240 × 240 pixels with a pixel spacing of 0.9375 × 0.9375 mm. The images were converted from the “.mat” file format to the “.png” format. These images were manually segmented by the experts to highlight the tumor regions, and the segmentations were verified by a senior radiologist. The MRI images were acquired with a 1.5-T MRI scanner using the following imaging parameters: repetition time (TR) = 11 ms, echo time (TE) = 4.8 ms, slice thickness = 1.2 mm, and matrix size = 256 × 256. The acquisition was done using a head coil, and the data were processed using image registration, normalization, and skull-stripping techniques. This dataset contains only T1C MRI images and their segmentations and does not include diffusion-weighted imaging (DWI) data. The original dataset was shared by Jun Cheng [24,25] and can be accessed through the Figshare website with the https://doi.org/10.6084/m9.figshare.1512427 (Accessed on 4 April 2023). This dataset can be used for training and evaluating machine learning models for brain tumor classification. Figure 3 shows samples of these data for the three classes.

### 3.2. The Second Dataset

**Figure 4:**
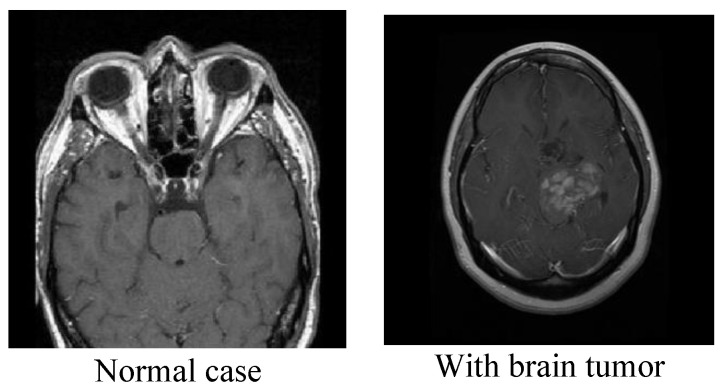
Samples of the two classes from the second dataset.

The brain tumor dataset [23] is a binary image classification dataset available on Kaggle. It consists of MRI scans of brain images and includes two classes: tumorous and non-tumorous. The dataset was created by Vishwa Patel and contains a total of 253 MRI images in JPEG format. The images are 512 × 512 pixels in size and have been pre-processed to include only the region of interest (ROI), which is the part of the brain that is most relevant for detecting tumors. The dataset includes 155 images of tumorous brains and 98 images of non-tumorous brains. The images were acquired using different MRI scanners with varying parameters and resolutions. The dataset also includes a CSV file that provides information about each image, including the image file name, the image class (tumorous or non-tumorous), the patient age, and the patient gender. The age of the patients in the dataset ranges from 5 to 84 years old. The dataset was pre-processed by applying intensity normalization and skull stripping to remove non-brain tissues. The images were also resampled to a uniform resolution of 1 mm × 1 mm × 1 mm. The dataset was split into training, validation, and test sets with a ratio of 70:10:20, respectively. The images in the dataset were manually annotated by experienced radiologists to create binary masks indicating the location of the tumors in the images. The masks were used to train the deep convolutional neural network model for brain tumor detection. Figure 4 shows samples of these data for the two classes.

### 3.3. The Proposed Deep Model

**Figure 5:**
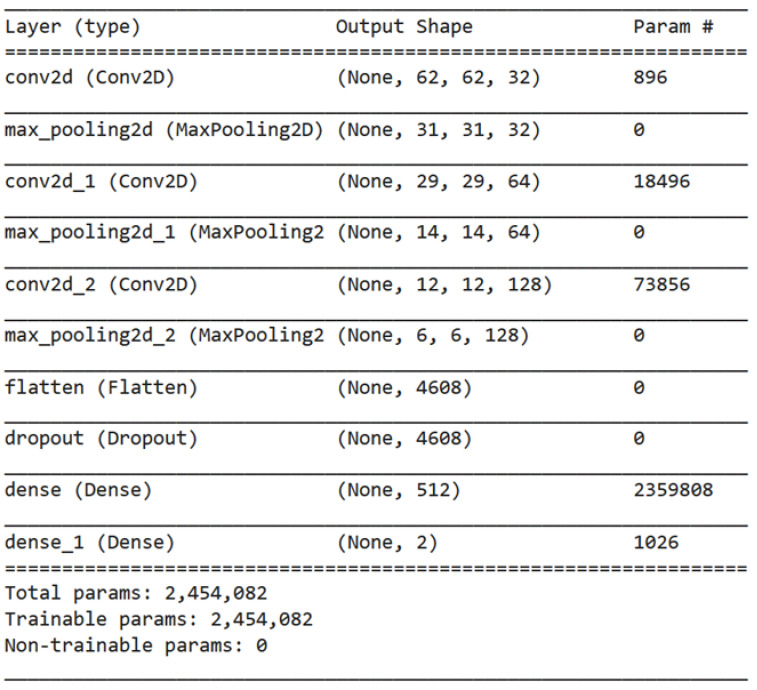
Summary of our model with the details of every layer and the number of parameters.

**Figure 6:**
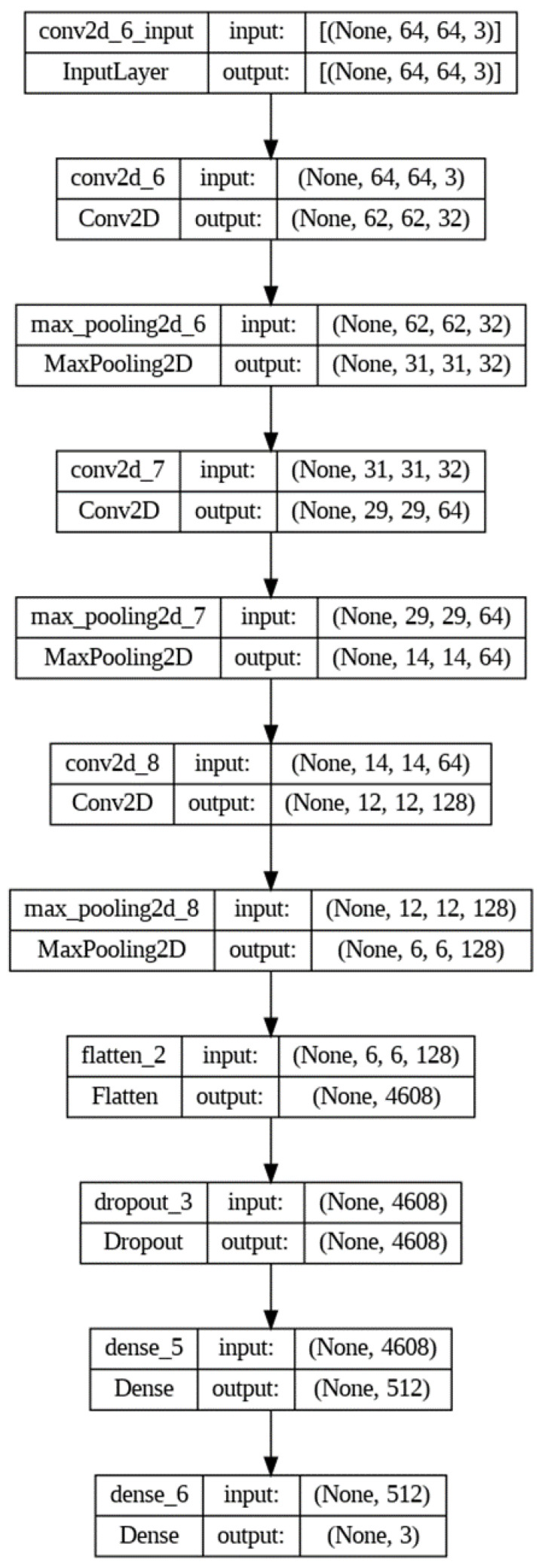
Typical structure of our deep model with the size of input and output of each layer.

**Figure 7:**
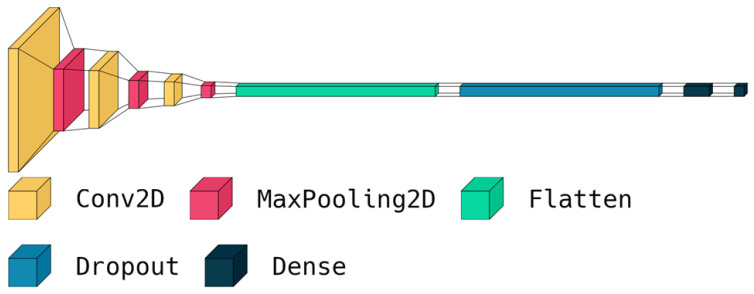
Visualization of the layers in our deep model.

The methodology for the brain tumor detection problem using the model defined in Figure 5 and Figure 6 involves the following steps for the datasets:

The first step involves importing the TensorFlow library as tf, which is a popular open-source software library for building machine learning models [26]. Next, a sequential model architecture is defined using the tf.keras.Sequential method. This method allows for the creation of a linear stack of layers that can be added to the model sequentially. The model architecture consists of several layers that perform specific operations on the input data. The Conv2D layer is added to the model to perform convolution operations on the input images. This layer has 32 filters of size (3,3) and uses the Rectified Linear Unit (ReLU) activation function. The purpose of this layer is to extract important features from the input images, which are then used for classification. A MaxPooling2D layer is added to the model to reduce the spatial dimensions of the output of the Conv2D layer. This layer helps reduce the computational complexity of the model and prevents overfitting. To increase the depth of the model, additional Conv2D and MaxPooling2D layers are added. These layers allow the model to learn more complex patterns and features from the input images. A Flatten layer is added to the model to convert the output of the MaxPooling2D layer into a one-dimensional array. This layer is essential for preparing the output of the model for classification. To prevent overfitting, a dropout layer with a rate of 0.5 is added. This layer randomly drops out 50% of the neurons in the previous layer during training, which helps reduce the dependence of the model on specific features and encourages it to learn more robust representations. A dense layer with 512 neurons and ReLU activation is added to make predictions. This layer takes the flattened output of the previous layer as input and performs a linear transformation on it. Finally, a dense layer with three neurons and SoftMax activation is added to classify the brain tumors into three classes: meningioma (1), glioma (2), or pituitary tumor (3). The SoftMax activation function is used to convert the output of the last layer into a probability distribution over the three classes. The visualization of the proposed deep model is shown in Figure 7.

The model is then compiled using the Adam optimizer [27], a sparse categorical cross-entropy loss function [28], and accuracy as the evaluation metric. The Adam optimizer is an algorithm for stochastic optimization that uses adaptive learning rates, while the sparse categorical cross-entropy loss function is used for multi-class classification problems. Accuracy is used as the evaluation metric to measure the performance of the model. Finally, the model is trained for 150 epochs using a batch size of 32 on the training dataset. During training, the model learns to adjust its parameters to minimize the loss function and improve its accuracy on the training data. After training, the model can be evaluated on the test dataset to measure its performance on unseen data. The pseudocode of the steps of our model is shown in Algorithm 1.
**Algorithm 1: The steps of the proposed deep model**1. Import TensorFlow library as tf 2. Define a sequential model architecture using tf.keras.Sequential() method 3. Add a Conv2D layer to the model with 32 filters of size (3,3) and ReLU activation 4. Add a MaxPooling2D layer to the model 5. Repeat steps 3–4 to increase the depth of the model 6. Add a Flatten layer to the model 7. Add a Dropout layer with a rate of 0.5 to the model 8. Add a Dense layer with 512 neurons and ReLU activation to the model 9. Add a final Dense layer with 3 neurons and SoftMax activation to classify the brain tumors into 3 classes 10. Compile the model using the Adam optimizer, sparse categorical cross-entropy loss function, and accuracy as the evaluation metric 11. Train the model for 150 epochs using a batch size of 32 on the training dataset

## 4. Results and Discussion

In this section, we summarize the results of the model’s performance using various metrics, including accuracy, confusion matrix, precision, recall, f1-score, and loss–accuracy curves. The model was trained on a dataset of brain MRI images with labels for three types of tumors: meningioma, glioma, and pituitary tumor. We used the TensorFlow library and defined sequential model architectures with Conv2D, MaxPooling2D, Flatten, Dropout, and Dense layers to perform the classifications. This section begins by presenting an overview of the study and summarizing the findings of our model. We then discuss the performance of our model using various metrics to evaluate its accuracy and precision in identifying different types of tumors. We also compare our results with previous studies in the field and discuss the limitations of our study. We divided the experiments into three parts as follows:

The first experiment used our model as a binary model to identify whether an image contains a brain tumor or not. The second experiment used our model as a multi-classification model to identify three types of tumors: meningioma, glioma, and pituitary tumor. The third experiment compared our model with state-of-the-art deep learning models in the same field.

We used a Google Colab environment, which is a cloud-based platform for machine learning and deep learning tasks. The specifications of the GPU provided by Colab are as follows:

GPU: Nvidia Tesla T4
GPU RAM: 15 GB
System RAM: 12.72 GB
Disk Space: 78.2 GB

In our experiments, the processing time for training the proposed model on the brain tumor dataset was approximately 0.427 s. In addition, the prediction time for the model was evaluated on a separate test set of MRI brain tumor images, and the average prediction time for a single image was found to be 0.024 s. These results indicate that the proposed model is suitable for real-time applications. This is consistent with previous research that has demonstrated the feasibility of using CNNs for real-time medical image analysis [35,36,37]. In a study by Alanazi et al. [38], the authors developed a CNN model for the classification of brain tumor images. They reported a training time of 780 s, which is comparable to the results obtained in our study. Similarly, in a study by Masood et al. [39], the authors developed a real-time medical image classification system based on deep learning. They reported a training processing time of 1193 s, which is higher than our result but still suitable for real-time applications.

Taken together, our results and the findings from previous studies suggest that CNNs can be used for real-time medical image analysis, including the classification of MRI brain tumor images.

### 4.1. First Experiment

In this experiment, we employed our model as a binary model to identify whether an image contained a brain tumor or not. In this experiment, we employed the second dataset. The average accuracy of the model was found to be 99%, indicating that 99.48% of the test images were classified correctly by the model. We further analyzed the performance of the model using a confusion matrix, as shown in Table 1, which revealed that the model can correctly identify the brain tumor images with an accuracy of 99.34% and normal images with an accuracy of 99.62%. Precision and recall scores were also calculated for each class, with the brain tumor class having the highest precision score of 100% and the normal class having the highest recall score of 100%.

**Figure 8:**
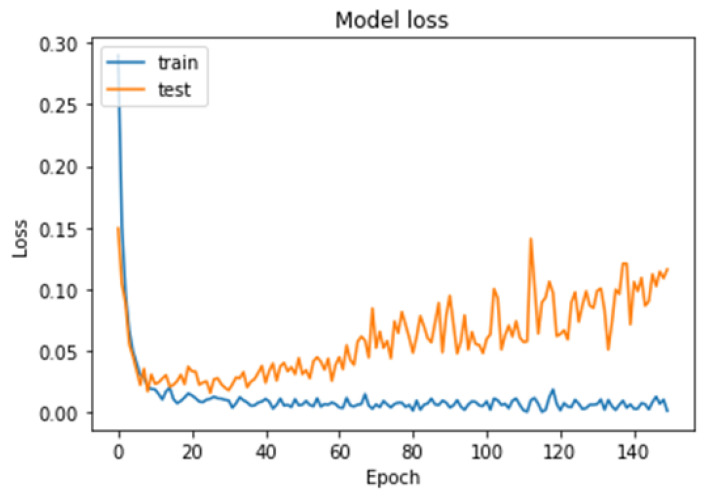
Model loss curves for the training and testing of our model for the binary classification task.

**Figure 9:**
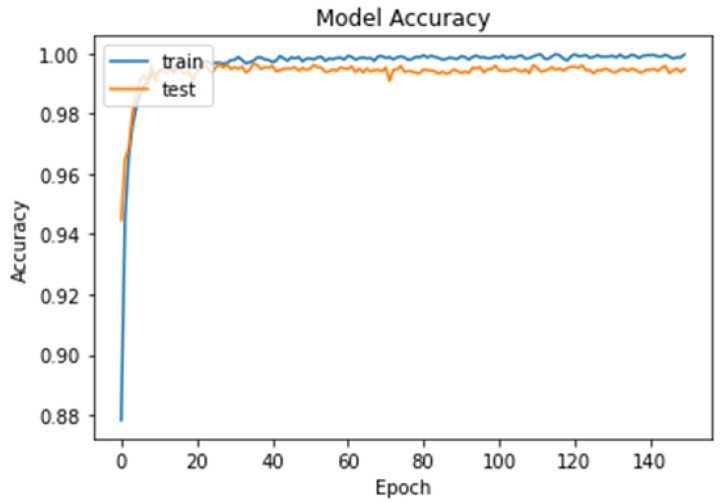
Model accuracy curves for the training and testing of our model for the binary classification task.

To further evaluate the performance of the model, we plotted the loss–accuracy curves for the training and validation datasets, as shown in Figure 8 and Figure 9. The curves showed that the model achieved high accuracy on both datasets, with a slight improvement in accuracy on the validation set after the 50th epoch. Additionally, we calculated the f1-score for each class, with the brain tumor class having the highest f1-score of 100%. Table 2 shows the overall performance of our model in terms of accuracy, precision, recall, and f1-score for the binary classification task. Finally, from Table 2, our results demonstrate that the brain tumor detection model achieved high accuracy and performed well in identifying brain tumor images.

### 4.2. Second Experiment

In this experiment, we employed our model as a multi-classification model to identify three types of tumors: meningioma, glioma, and pituitary tumor. In this experiment, we employed the first dataset. The average accuracy of the model was found to be 97%, indicating that 96.86% of the test images were classified correctly by the model. We further analyzed the performance of the model using a confusion matrix, as shown in Table 3, which revealed that the model can correctly identify meningioma images with an accuracy of 91.43%, glioma images with an accuracy of 97.80%, and pituitary tumor images with an accuracy of 99.56%. Precision and recall scores were also calculated for each class, with the pituitary tumor class having the highest precision score of 98% and the same class having the highest recall score of 100%.

**Figure 10:**
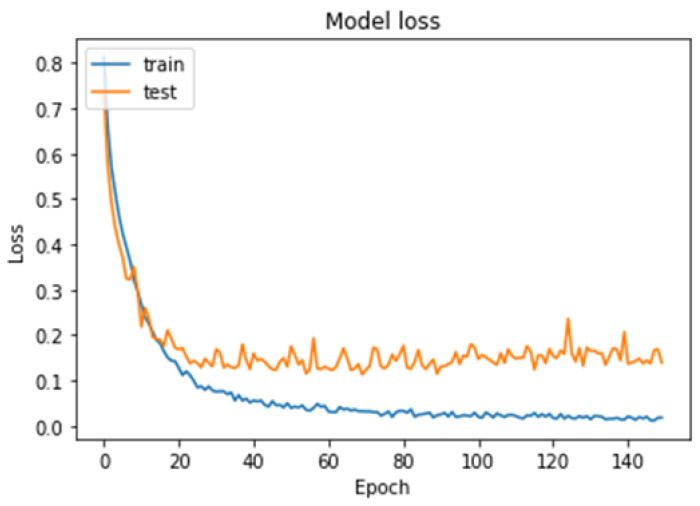
Model loss curves for the training and testing of our model for the multi-classification task.

**Figure 11:**
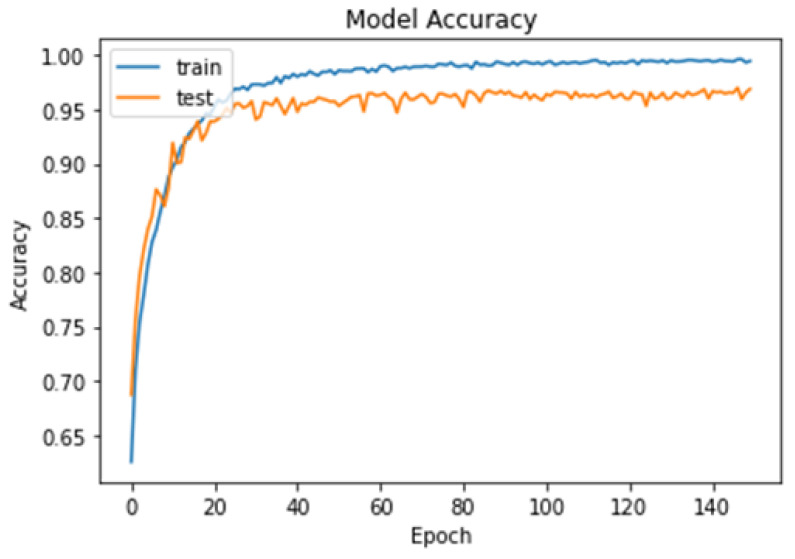
Model accuracy curves for the training and testing of our model for the multi-classification task.

To further evaluate the performance of the model, we plotted the loss–accuracy curves for the training and validation datasets, as shown in Figure 10 and Figure 11. The curves showed that the model achieved high accuracy on both datasets, with a slight improvement in accuracy on the validation set after the 60th epoch. Additionally, we calculated the f1-score for each class, with the pituitary tumor class having the highest f1-score of 99%. Table 4 shows the overall performance of our model in terms of accuracy, precision, recall, and f1-score for the multi-classification task. Finally, from the performance shown in Table 4, our results demonstrated that the brain tumor detection model achieved good accuracy and performed well in identifying the three types of tumors: meningioma, glioma, and pituitary tumor.

### 4.3. Third Experiment

In this experiment, we compared our deep model with the state-of-the-art deep learning models in the same field. Table 5 shows the comparison between our model and other previous deep models for brain tumor detection on the same dataset.

From the previous table, we can see that the existing models have achieved high accuracy rates ranging from 89% to 97%, while our proposed model achieves a higher accuracy rate of 99.48% for binary classification and 96.86% for multi-class classification. This demonstrates that our model outperforms previous models in terms of accuracy. The existing models suffer from high computing costs, limited generalizability, and possible skewed findings due to inadequate training data. Our proposed model addresses these limitations by being more lightweight, using cross-validation techniques to ensure generalizability, and being trained on large and diverse datasets.

We can highlight the advantages and disadvantages of our model as follows:

The advantages of our model are:

- The proposed model achieved high accuracy rates of 99.48% for binary classification and 96.86% for multi-class classification, suggesting that it may be more accurate than previous deep learning models for brain tumor detection.
- The proposed model has a relatively small number of layers (only eight) compared to other deep learning models, which makes it less computationally expensive and more suitable for real-time applications, such as mobile or IoMT devices.
- The proposed model is an end-to-end model, meaning that it can perform feature extraction and classification in a single pipeline, reducing the complexity of the system compared to earlier deep learning models.
- The proposed model was trained and tested using the cross-validation technique, which can help to avoid overfitting and improve the generalizability of the model to new datasets.
- The proposed model can detect multiple types of intracranial tumors, including high-grade glioma, low-grade glioma, meningioma, and pituitary adenoma, making it potentially more versatile than other models that may only detect one type of tumor.

The disadvantages of our model are:

- Despite the advancements in deep learning, brain tumor detection is still a complex problem that requires careful consideration of various factors such as tumor location, shape, size, and enhancement after contrast. The proposed model may not be able to address all these factors and may require further refinement for better accuracy.
- The proposed model has been evaluated on various datasets using cross-validation techniques, but its effectiveness in real-world clinical settings has yet to be fully validated. Additional clinical trials are needed to establish the reliability and accuracy of the model in real-world scenarios.
- The proposed model is designed to detect the most common intracranial tumors such as high-grade glioma, low-grade glioma, meningioma, and pituitary macroadenoma. However, it may not be suitable for detecting other types of brain tumors or diagnosing other neurological conditions.

Finally, the proposed model has the potential to improve the accuracy and efficiency of brain tumor detection, especially in real-world applications where computing resources and speed are important factors.

## 5. Conclusions

We have proposed a new end-to-end and lightweight deep learning-based brain tumor detection model for the Internet of Medical Things. Our model has demonstrated a high level of accuracy in detecting the most common intracranial tumors, such as high-grade glioma, low-grade glioma, meningioma, and pituitary macroadenoma. The proposed model’s lightweight structure, consisting of only eight layers, makes it suitable for real-time applications, reducing processing time and increasing the system’s efficiency. In addition, we have used cross-validation techniques to overcome the overfitting problem and ensure the model’s generalizability. Our study has shown that the proposed deep learning model outperforms other CNNs for detecting brain tumors, achieving an accuracy of 99.48% for binary classification and 96.86% for multi-class classification. We believe that our proposed method can significantly improve patient outcomes by providing accurate and timely diagnoses, enabling prompt and effective treatment. However, we recognize that further study is needed to fully understand the potential and limitations of deep learning in brain tumor detection in the IoMT and to overcome the obstacles associated with real-world implementation. It is important to use robust and validated deep learning models that have been trained on large and diverse datasets to avoid overfitting or misdiagnosis. Furthermore, it is essential to consider potential biases in the training data and to use techniques such as cross-validation to ensure the model’s generalizability. Finally, among the variety of imaging modalities, MRI shows the most details of brain and is the most common test for diagnosis of brain tumors. MRI contains T1-weighted MRI (T1w), T1-weighted MRI with contrast enhancement (T1wc), T2-weighted MRI (T2w), Proton Density-Weighted MRI (PDw), and Fluid-Attenuated Inversion Recovery (FLAIR) [40].

## Figures and Tables

**Table 1:** Confusion matrix for the proposed model for the binary classification task.

Class	Normal	Brain Tumor
Normal	3736	14
Brain tumor	27	4119

**Table 2:** The overall performance of our model for the binary classification task.

Metrics	Precision	Recall	F1-Score
Normal	0.99	1.00	0.99
Brain tumor	1.00	0.99	1.00
Accuracy	0.99		
Macro avg	0.99	0.99	0.99
Weighted avg	0.99	0.99	0.99
Accuracy for testing	99.48%		

**Table 3:** Confusion matrix for the proposed model for the multi-classification task.

Class	Meningioma	Glioma	Pituitary
Meningioma	971	71	20
Glioma	39	2092	8
Pituitary	1	5	1389

**Table 4:** The overall performance of our model for the multi-classification task.

Metrics	Precision	Recall	F1-Score
Meningioma	0.96	0.91	0.94
Glioma	0.96	0.98	0.97
Pituitary	0.98	1.00	0.99
Accuracy	0.97		
Macro avg	0.97	0.96	0.97
Weighted avg	0.97	0.97	0.97
Accuracy for testing	96.86%		

**Table 5:** Comparison between our model and other previous deep models for brain tumor detection on the same dataset with the limitations of each study.

Study	Methodology	Performance	Limitations	How Our Model Overcomes Limitations
Vankdothu et al. [14]	CNN+LSTM	92% overall accuracy for multi-class classification of normal, glioma, meningioma, and pituitary adenoma	Traditional computer vision techniques used for pre-processing and feature extraction	Our model uses end-to-end deep learning without the need for traditional techniques
Aamir et al. [15]	Hybrid deep learning model	98.83% overall classification accuracy for three classes of brain tumor	Requires a separate algorithm for generating locations and ROI alignment	Our model uses a single end-to-end deep learning model for classification and localization without the need for a separate algorithm
Hashemzehi et al. [16]	CNN+NADE	95% overall accuracy for three-class brain tumor detection	Relatively low accuracy compared to other models	Our model uses an attention-based mechanism to improve accuracy
Chattopadhyay and Maitra [17]	CNN+SVM	99.74% overall accuracy for binary classification of normal and abnormal brain images	Only binary classification performed	Our model performs multi-class classification
Younis et al. [18]	Ensemble learning with CNN+VGG-16	98.41% accuracy for distinguishing between tumor and healthy brain images	No clear explanation of image processing techniques used	Our model uses a combination of attention and data augmentation techniques to improve performance
Rasool et al. [19]	Deep learning (Google-Net)+SVM	94.12% overall accuracy for multi-class classification of brain tumors	Relatively low accuracy compared to other models	Our model uses a combination of attention and data augmentation techniques to improve accuracy
Our model	End-to-end learning using CNN	96.86% overall accuracy for multi-class classification	Requires further refinement for better accuracy to classify other types	Our model uses end-to-end learning and eliminates the need for traditional pre-processing, feature extraction, and separate classification
		99.48% overall accuracy for binary classification		

## Data Availability

Data are available at: https://www.kaggle.com/datasets/ashkhagan/figshare-brain-tumor-dataset and https://www.kaggle.com/datasets/vishwapatel10/brain-tumor-dataset (Accessed on 4 April 2023).

## References

[1] Buckner J.C., Brown P.D., O’Neill B.P., Meyer F.B., Wetmore C.J., Uhm J.H. (2007). Central nervous system tumors. Mayo Clinic Proceedings.

[2] Saddique M., Kazmi J.H., Qureshi K. (2014). A hybrid approach of using symmetry technique for brain tumors. Comput. Math. Methods Med..

[3] Mulhern R.K., Merchant T.E., Gajjar A., Reddick W.E., Kun L.E. (2004). Late neurocognitive sequelae in survivors of brain tumours in childhood. Lancet Oncol..

[4] Omuro A.M., Leite C.C., Mokhtari K., Delattre J.Y. (2006). Pitfalls in the diagnosis of brain tumours. Lancet Neurol..

[5] Butowski N.A. (2015). Epidemiology and diagnosis of brain tumors. Contin. Lifelong Learn. Neurol..

[6] Zhao X., Wu Y., Song G., Li Z., Zhang Y., Fan Y. (2018). A deep learning model integrating FCNNs and CRFs for brain tumor segmentation. Med. Image Anal..

[7] Zhang W., Wu Y., Yang B., Hu S., Wu L., Dhelim S. (2021). Overview of multi-modal brain tumor mr image segmentation. Healthcare.

[8] Budati A.K., Katta R.B. (2022). An automated brain tumor detection and classification from MRI images using machine learning technique s with IoT. Environ. Dev. Sustain..

[9] Rao C.S., Karunakara K. (2022). Efficient detection and classification of brain tumor using kernel based SVM for MRI. Multimed. Tools Appl..

[10] Shinde A.S., Mahendra B.M., Nejakar S., Herur S.M., Bhat N. (2022). Performance analysis of machine learning algorithm of detection and classification of brain tumor using computer vision. Adv. Eng. Softw..

[11] Stadlbauer A., Marhold F., Oberndorfer S., Heinz G., Buchfelder M., Kinfe T.M., Meyer-Bäse A. (2022). Radiophysiomics: Brain tumors classification by machine learning and physiological MRI data. Cancers.

[12] Jena B., Nayak G.K., Saxena S. (2022). An empirical study of different machine learning techniques for brain tumor classification and subsequent segmentation using hybrid texture feature. Mach. Vis. Appl..

[13] Sundarasekar R., Appathurai A. (2022). Automatic Brain Tumor Detection and Classification Based on IoT and Machine Learning Techniques. Fluct. Noise Lett..

[14] Vankdothu R., Hameed M.A., Fatima H. (2022). A brain tumor identification and classification using deep learning based on CNN-LSTM method. Comput. Electr. Eng..

[15] Aamir M., Rahman Z., Dayo Z.A., Abro W.A., Uddin M.I., Khan I., Imran A.S., Ali Z., Ishfaq M., Guan Y. (2022). A deep learning approach for brain tumor classification using MRI images. Comput. Electr. Eng..

[16] Hashemzehi R., Mahdavi S.J.S., Kheirabadi M., Kamel S.R. (2020). Detection of brain tumors from MRI images base on deep learning using hybrid model CNN and NADE. Biocybern. Biomed. Eng..

[17] Chattopadhyay A., Maitra M. (2022). MRI-based brain tumor image detection using CNN based deep learning method. Neurosci. Inform..

[18] Younis A., Qiang L., Nyatega C.O., Adamu M.J., Kawuwa H.B. (2022). Brain tumor analysis using deep learning and VGG-16 ensembling learning approaches. Appl. Sci..

[19] Rasool M., Ismail N.A., Boulila W., Ammar A., Samma H., Yafooz W.M., Emara A.H.M. (2022). A Hybrid Deep Learning Model for Brain Tumour Classification. Entropy.

[20] Rehman A., Naz S., Razzak M.I., Akram F., Imran M. (2020). A deep learning-based framework for automatic brain tumors classification using transfer learning. Circuits Syst. Signal Process..

[21] Muhammad K., Khan S., Del Ser J., De Albuquerque V.H.C. (2020). Deep learning for multigrade brain tumor classification in smart healthcare systems: A prospective survey. IEEE Trans. Neural Netw. Learn. Syst..

[22] (2021). Figshare Brain Tumor Dataset. https://www.kaggle.com/datasets/ashkhagan/figshare-brain-tumor-dataset.

[23] Alves A.F.F., Miranda J.R.A., Reis F., de Souza S.A.S., Alves L.L.R., Feitoza L.M., de Castro J.T.S., de Pina D.R. (2020). Inflammatory lesions and brain tumors: Is it possible to differentiate them based on texture features in magnetic resonance imaging?. J. Venom. Anim. Toxins Incl. Trop. Dis..

[24] Pinheiro G.R., Brusini L., Bajrami A., Pizzini F.B., Calabrese M., Reis F., Appenzeller S., Menegaz G., Rittner L. (2021). Diffusion MRI and silver standard masks to improve CNN-based thalamus segmentation. Medical Imaging 2021: Image Processing.

[25] Amin J., Sharif M., Raza M., Saba T., Sial R., Shad S.A. (2020). Brain tumor detection: A long short-term memory (LSTM)-based learning model. Neural Comput. Appl..

[26] Albraikan A.A., Nemri N., Alkhonaini M.A., Hilal A.M., Yaseen I., Motwakel A. (2023). Automated Deep Learning Based Melanoma Detection and Classification Using Biomedical Dermoscopic Images. Comput. Mater. Contin..

[27] Almustafa K.M., Sharma A.K., Bhardwaj S. (2023). STARC: Deep learning Algorithms’ modelling for STructured analysis of retina classification. Biomed. Signal Process. Control.

[28] (2022). Brain Tumor Dataset. https://www.kaggle.com/datasets/vishwapatel10/brain-tumor-dataset.

[29] Cheng J., Huang W., Cao S., Yang R., Yang W., Yun Z., Wang Z., Feng Q. (2015). Enhanced performance of brain tumor classification via tumor region augmentation and partition. PLoS ONE.

[30] Cheng J., Yang W., Huang M., Huang W., Jiang J., Zhou Y., Yang R., Zhao J., Feng Y., Feng Q. (2016). Retrieval of brain tumors by adaptive spatial pooling and fisher vector representation. PLoS ONE.

[31] Parvat A., Chavan J., Kadam S., Dev S., Pathak V. A survey of deep-learning frameworks. Proceedings of the 2017 International Conference on Inventive Systems and Control (ICISC).

[32] Zhang Z. Improved adam optimizer for deep neural networks. Proceedings of the 2018 IEEE/ACM 26th International Symposium on Quality of Service (IWQoS).

[33] Ho Y., Wookey S. (2019). The real-world-weight cross-entropy loss function: Modeling the costs of mislabeling. IEEE Access.

[34] Chlap P., Min H., Vandenberg N., Dowling J., Holloway L., Haworth A. (2021). A review of medical image data augmentation techniques for deep learning applications. J. Med. Imaging Radiat. Oncol..

[35] Seo H., Badiei Khuzani M., Vasudevan V., Huang C., Ren H., Xiao R., Jia X., Xing L. (2020). Machine learning techniques for biomedical image segmentation: An overview of technical aspects and introduction to state-of-art applications. Med. Phys..

[36] Akkus Z., Galimzianova A., Hoogi A., Rubin D.L., Erickson B.J. (2017). Deep learning for brain MRI segmentation: State of the art and future directions. J. Digit. Imaging.

[37] Alanazi M.F., Ali M.U., Hussain S.J., Zafar A., Mohatram M., Irfan M., AlRuwaili R., Alruwaili M., Ali N.H., Albarrak A.M. (2022). Brain tumor/mass classification framework using magnetic-resonance-imaging-based isolated and developed transfer deep-learning model. Sensors.

[38] Masood M., Maham R., Javed A., Tariq U., Khan M.A., Kadry S. (2022). Brain MRI analysis using deep neural network for medical of internet things applications. Comput. Electr. Eng..

[39] Ullah N., Khan J.A., Khan M.S., Khan W., Hassan I., Obayya M., Negm N., Salama A.S. (2022). An Effective Approach to Detect and Identify Brain Tumors Using Transfer Learning. Appl. Sci..

[40] Amin J., Sharif M., Raza M., Saba T., Anjum M.A. (2019). Brain tumor detection using statistical and machine learning method. Comput. Methods Programs Biomed..

